# A Retrospective Study on Amoxicillin Susceptibility in Severe *Haemophilus influenzae* Pneumonia

**DOI:** 10.1155/2020/2093468

**Published:** 2020-09-09

**Authors:** Pierre Danneels, Maria Concetta Postorino, Alessio Strazzulla, Nabil Belfeki, Aurelia Pitch, Frank Pourcine, Sebastien Jochmans, Vincent Dubée, Mehran Monchi, Sylvain Diamantis

**Affiliations:** ^1^Infectious Diseases Unit, Groupe Hospitalier Sud Ile de France, Melun, France; ^2^Internal Medicine Unit, Groupe Hospitalier Sud Ile de France, Melun, France; ^3^Medical Biology Laboratory, Groupe Hospitalier Sud Ile de France, Melun, France; ^4^Intensive Care Unit, Groupe Hospitalier Sud Ile de France, Melun, France; ^5^CRCINA, Inserm, Paris, France; ^6^Equipe ATIP AVENIR, CRCINA, Inserm, Université de Nantes, Université d'Angers, Angers, France; ^7^Service de Maladies Infectieuses et Tropicales, CHU Angers, Angers, France

## Abstract

**Introduction:**

Treatment of *Haemophilus influenzae* (Hi) pneumonia is on concern because resistance to amoxicillin is largely diffused. This study describes the evolution of resistance to amoxicillin and amoxicillin/clavulanic acid (AMC) in Hi isolates and characteristics of patients with Hi severe pneumonia.

**Methods:**

A monocentric retrospective observational study including patients from 2008 to 2017 with severe pneumonia hospitalized in ICU. Evolution of amoxicillin and AMC susceptibility was showed. Characteristics of patients with Hi pneumonia were compared to characteristics of patients with *Streptococcus pneumoniae* (Sp) pneumonia, as reference. Risk factors for amoxicillin resistance in Hi were investigated.

**Results:**

Overall, 113 patients with Hi and 132 with Sp pneumonia were included. The percentages of AMC resistance among Hi strains decreased over the years (from 10% in 2008-2009 to 0% in 2016-2017) while resistance to amoxicillin remained stable at 20%. Also, percentages of Sp resistant strains for amoxicillin decreased over years (from 25% to 3%). Patients with Hi pneumonia experienced higher prevalence of bronchitis (18% vs. 8%, *p*=0.02, chronic obstructive pulmonary disease (43% vs. 30% *p*=0.03), HAP (18% vs. 7%, *p*=0.01, ventilator-associated pneumonia (27% vs. 17%, *p*=0.04, and longer duration of mechanical ventilation (8 days vs. 6 days, *p*=0.04) than patients with Sp pneumonia. Patients with Sp pneumonia had more frequently local complications than patients with Hi pneumonia (17% vs. 7%, *p*=0.03). De-escalation of antibiotics was more frequent in patients with Sp than in patients with Hi (67% vs. 53%, *p*=0.03). No risk factors were associated with amoxicillin resistance among patients with Hi pneumonia.

**Conclusions:**

Amoxicillin resistance was stable over time, but no risk factors were detected. AMC resistance was extremely low, suggesting that AMC could be used for empiric treatment of Hi pneumonia, as well as other molecules, namely, cephalosporins. Patients with Hi pneumonia had more pulmonary comorbidities and severe diseases than patients with Sp pneumonia.

## 1. Introduction

*Haemophilus influenzae* (Hi) is one of the most frequent causes of community-acquired pneumonia (CAP) [1], but its isolation is frequent also during hospital-acquired pneumonia (HAP) [2]. Antibiotic treatment of severe pneumonia due to Hi infection is on concern, due to the spreading of antimicrobial resistances. Indeed, the high *β*-lactamase production causes amoxicillin resistance in many Hi strains and, for this reason, third-generation cephalosporins (3GC) are frequently prescribed for empiric treatment [3–5].

Current politics of antimicrobial stewardship discourage the use of 3GC because these molecules enhance the selection of extended-spectrum *β*-lactamases (ESBL), namely, cephalosporinases. Also, 3GC did not show superiority in terms of mortality when they were compared to amoxicillin/clavulanic acid (AMC) for the treatment of CAP [6]. Moreover, other factors should also be considered for the choice of the antibiotic treatment, including patient's characteristics, site of infection, severity of the disease, and local resistance epidemiology [7, 8].

The objective of this study was to evaluate the evolution of resistance to amoxicillin and AMC of Hi isolates during pneumonia and clinical characteristics of patients hospitalized in intensive care unit (ICU) with Hi-associated pneumonia.

## 2. Methods

We conducted a monocentric retrospective observational study in a 350-bed general hospital in the Ile-de-France region in France, including adult patients admitted in ICU from January 2008 to December 2017 with diagnosis of pneumonia and positive bacterial isolate for Hi or *Streptococcus pneumoniae* (Sp) pneumonia. Exclusion criteria extrapulmonary infection without pneumonia, colonization without diagnosis of pneumonia, and chronic obstructive pulmonary disease (COPD) exacerbations without evidence of parenchyma infiltrates.

This study was conducted in accordance with the Declaration of Helsinki and national and institutional standards. The local institutional review board did not waive patients' consent obligation due to the retrospective character of the study and since this study did not require neither further laboratory analysis nor different clinical management compared to daily clinical routine.

The diagnosis of pneumonia was posed by the intensive care specialist basing on clinical and radiological data and according to international guidelines [9, 10]. Both CAP and HAP were included in the analysis. Recurrences of pneumonia in the same patients were excluded from the analysis. Data about patients' characteristics, laboratory analyses, and treatment outcomes were gathered from software used in daily clinical practice: Sillage v15.5.1.22 and CGM Lab channel 1.20.33686.

For each patient, demographical characteristics, medical history, predisposing risk factor for pneumonia, comorbidities, type of pneumonia, kind of samples, factor associated with severity of pneumonia, antimicrobial treatment de-escalation, and outcome (death) were analyzed.

All positive isolates for Hi and Sp were considered. Samples included blood culture, pleural fluid, cerebrospinal fluid, and bronchial secretion (sputum in patients able to cough or endotracheal aspiration in ventilated patients). The results of pneumococcal urinary antigen test were not considered.

Bronchial secretions were analyzed if they fulfilled 4 and 5 Bartlett criteria (<25 squamous epithelial cells and >25 leukocytes per low-power field). The significant threshold for the bacterial culture was 10^7^ CFU/ml for sputum, 10^5^ for endotracheal aspirate, 10^4^ for bronchoalveolar lavage, and 10^3^ for transbronchial biopsy.

Minimal inhibitory concentrations (MICs) were obtained by diffusion on agar disk. During the period, 2005 and 2013 European Committee on Antimicrobial Susceptibility Testing (EUCAST) guidelines were used to interpret resistance results [11]. During the period, no modification of clinical breakpoints occurred. All strains with intermediate or resistant MIC were considered resistant.

For amoxicillin resistance in Hi, penicillinases were detected by Cefinase disk procedure. For Sp, susceptibility test to AMC was not performed because resistance to amoxicillin is due to the modification of penicillin-binding protein rather than *β*-lactamase production. Therefore, no advantage is expected in treatment with AMC in case of Sp strains resistant to amoxicillin.

Data were analyzed using Epi Info 7.0 statistical software. Continuous data were expressed as median ± interquartile range (IQR) and were compared using the Kruskal–Wallis test. Categorical variables, expressed as percentages, were evaluated using a two-sided Fisher's exact test, as appropriate. Nominative significance was set at *p* < 0.05.

The following analysis were made: (i) description of the evolution of amoxicillin and AMC resistance in Hi and amoxicillin resistance in Sp; (ii) comparison of characteristics of patients with Hi and Sp pneumonia; (iii) comparison of characteristics of patients with Hi pneumonia according to amoxicillin resistance or susceptibility.

## 3. Results

**Figure 1:**
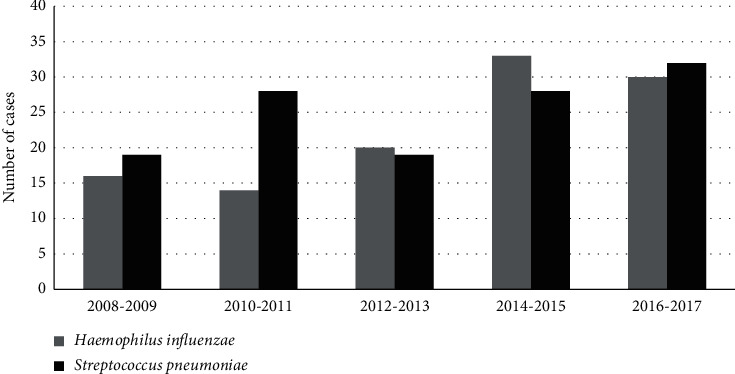
Evolution of diagnosis of *Streptococcus pneumoniae* and *Haemophilus influenzae* pneumonia in intensive care unit from 2008 to 2017. *Note*. Bed places in ICU implemented in 2014 causing an increase in the number of hospitalizations.

Overall, 311 positive samples were collected. After the application of exclusion criteria, 245/311 (79%) cases of pneumonia were included, 113/245 (46%) Hi and 132/245 (54%) Sp pneumonia.  Figure 1 shows the evolution of the number of patients admitted in ICU with diagnosis of Hi and Sp pneumonia from 2008 to 2017.

**Figure 2:**
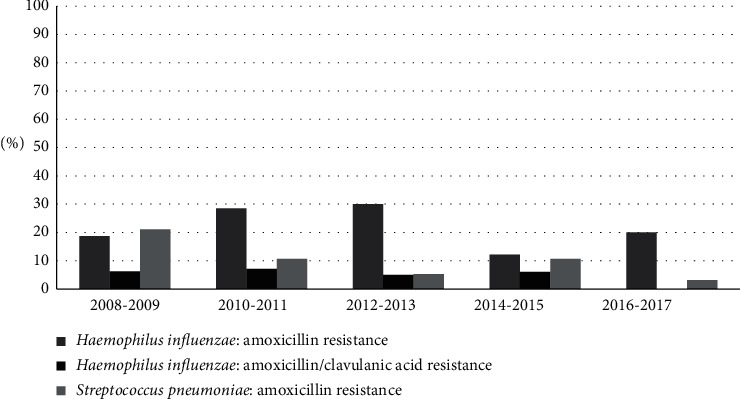
Evolution of resistance to amoxicillin and amoxicillin/clavulanic acid in *Haemophilus influenzae* and *Streptococcus pneumoniae*.

Hi strains had a stable resistance profile (over 20% strains in all periods observed) for amoxicillin; at the same time, resistance percentages for amoxicillin/clavulanic acid (AMC) decreased over the years (from 10% in 2008-2009 to 0% in 2016-2017, *p*=0.15). In Sp, amoxicillin resistance decreased over years (from 25% to 3%, *p* < 0.001).  Figure 2 shows the evolution of resistance to amoxicillin and AMC in Hi and Sp isolates.

When patients' characteristics were considered, patients with Hi-associated pneumonia had a higher prevalence of bronchitis (18% vs. 8%, *p*=0.02) and COPD (43% vs. 30% *p*=0.03) than patients with Sp-associated pneumonia. Also, patients with Hi-associated pneumonia had higher rates of HAP (18% vs. 7%, *p*=0.01), ventilator-associated pneumonia or VAP (27% vs. 17%, *p*=0.04), and a longer duration of mechanical ventilation (8 days vs. 6 days, *p*=0.04) while patients with Sp-associated pneumonia had more frequently local complications (17% vs. 7%, *p*=0.03). De-escalation of antimicrobial therapy was more frequent in patients with Sp than in patients with Hi pneumonia (67% vs. 53%, *p*=0.03). No other significant differences in clinical characteristics were reported (Table 1).

Among patients with Hi pneumonia, no statistical difference was detected when characteristics of patients with Hi isolates resistant or susceptible to amoxicillin were compared (Table 2).

## 4. Discussion

This study evaluated the susceptibility to amoxicillin and AMC of Hi isolated from patients hospitalized in ICU with severe pneumonia, and it showed that 80% of Hi strains were susceptible to amoxicillin. The relatively low rate of resistance to amoxicillin reflects a tendency of Hi to maintain amoxicillin susceptibility when involved in severe disease, as already showed by other authors [12].

In our study, a hypothetic empiric therapy with AMC would have covered 98% of microbiological strains of Hi. Considering the low rate of amoxicillin resistance of Sp in France (2.4%), an empirical treatment with AMC associated with another molecule for the treatment of intracellular agents (i.e., macrolide) could be reasonably prescribed in patients with suspected Hi or Sp pneumonia to cover both germs, at least for the treatment of CAP [13].

AMC is competitive with 3GC at least when antibiotic susceptibility of Hi (and Sp) is considered. However, the risk of ESBL selection is high with AMC as well as 3GC (cephalosporinases) and the ecologic impact of these molecules is far from being well explained. Thus, it is not possible to unequivocally state the best empiric regimen (AMC vs. 3GC) for pneumonia in terms of collateral damage.

On the other side, amoxicillin could have theoretically treated 80% of infection due to Hi and 97% of Sp infection by the end of 2017. However, our study failed to detect any factor associated with amoxicillin resistance. Therefore, it is not possible to propose amoxicillin for the treatment of severe pneumonia in patients hospitalized in ICU before a microbiological diagnosis (empirical treatment).

Both amoxicillin and AMC could be used as de-escalation, in order to spare molecules more susceptible of selecting resistant strains [14–16]. In our study, we found that de-escalation of antimicrobial therapy was prescribed more frequently in patients with Sp-associated pneumonia than in patients with Hi (67% vs. 53%, *p*=0.03), probably because patients with Hi infection had more severe clinical characteristics and comorbidities than patients with Sp infection. This could have enhanced clinicians to pursue a treatment with broad-spectrum antibiotics even after microbiologic diagnosis. Further studies should investigate factors which influence the choice of keeping broad-spectrum antibiotics rather than streamlining (de-escalation) toward narrow-spectrum molecules.

Hi pneumonia frequently developed in patients with pulmonary diseases and often it was healthcare-associated. Indeed, COPD, recent bronchitis, HAP, and VAP occurred more frequently among patients with Hi-associated pneumonia than among patients with Sp-associated pneumonia. Likely, the higher incidence of VAP was due to the longer duration of mechanical ventilation in patients with Hi infection. However, no radiologic difference was reported between Hi and Sp pneumonia. Our data suggest that patients with Hi-associated pneumonia were more likely to be admitted in ICU with a severe pulmonary disease than patients with Sp-associated pneumonia. Further efforts are required to prevent Hi pneumonia among patients with COPD, together with yearly vaccinations campaign for flu and Sp [17, 18].

Our results are limited by the retrospective observational nature of the study and by the monocentric design. However, our data are sufficiently appealing to provoke further studies in order to improve the clinical and microbiological approach to severe Hi-associated pneumonia. In particular, further prospective studies are required to compare the efficacy of empirical AMC (plus a macrolide) versus empirical 3GC (plus a macrolide) and the efficacy of de-escalation towards amoxicillin (from 3GC or AMC including regimens).

## 5. Conclusions

In conclusion, our study reported a decreasing resistance for amoxicillin and AMC among Hi isolates obtained from patients with severe pneumonia. AMC could be prescribed in patients with severe pneumonia to treat both Hi and Sp infections, as empirical treatment, while amoxicillin could be prescribed for de-escalation in 80% of Hi pneumonia. Patients with Hi had more pulmonary comorbidities and severe diseases than patients with Sp-associated pneumonia.

## Figures and Tables

**Table 1:** Patient's demographical, microbiological, and clinical characteristics.

Criteria	*Haemophilus influenzae* (*n* = 113)	*Streptococcus pneumoniae* (*n* = 132)	*p* value
*Demographical characteristics*			
Female gender, *n* (%)	46 (41)	53 (40)	1.000
Age, median (IQR)	64 (54–75)	61 (52–71)	0.141
BMI, median (IQR)	25 (22–31)	25 (22–28)	0.400
*Medical history*			
Recent bronchitis, *n* (%)	21 (19)	11 (8)	0.022
Previous pneumonia, *n* (%)	63 (56)	80 (61)	0.516
*Risk factors*			
Living in nursing home, *n* (%)	17 (15)	23 (17)	0.729
Smoking, *n* (%)	55 (49)	59 (45)	0.608
Alcohol abuse, *n* (%)	33 (29)	44 (33)	0.494
*Comorbidities*			
Immunosuppression, *n* (%)	17 (15)	23 (17)	0.729
Diabetes, *n* (%)	25 (22)	24 (18)	0.522
Cirrhosis, *n* (%)	9 (8)	10 (8)	1.000
Renal chronic failure, *n* (%)	5 (4)	5 (4)	1.000
Cardiac chronic failure, *n* (%)	21 (19)	27 (20)	0.749
COPD, *n* (%)	49 (43)	39 (30)	0.032
*Type of pneumonia*			
CAP, *n* (%)	93 (82)	123 (93)	0.010
HAP, *n* (%)	20 (18)	9 (7)	0.010
VAP, *n* (%)	15 (13)	6 (5)	0.021
Inhalation associated, *n* (%)	42 (37)	49 (37)	1.000
Meningitis associated, *n* (%)	0 (0)	4 (3)	0.126
Coinfection, *n* (%)	31 (27)	23 (17)	0.065
*Kind of sample*			
Pulmonary sample, *n* (%)	106 (94)	93 (70)	<0.001
Blood culture, *n* (%)	7 (6)	37 (28)	<0.001
Pleural fluid, *n* (%)	0 (0)	8 (6)	0.008
Cerebrospinal fluid, *n* (%)	0 (0)	4 (3)	0.126
*Severity of pneumonia*			
SAPS-II, median (IQR)	48 (33–62)	48 (35–58)	0.558
Local complication, *n* (%)	8 (7)	22 (17)	0.030
Organ failure, *n* (%)	108 (96)	128 (97)	0.736
ARDS, *n* (%)	17 (15)	23 (17)	0.729
MV, *n* (%)	107 (95)	119 (90)	0.234
OTI, *n* (%)	96 (85)	106 (80)	0.401
VAP during hospitalization, *n* (%)	31 (27)	22 (17)	0.044
Acute renal failure, *n* (%)	55 (49)	66 (50)	0.898
Dialysis, *n* (%)	7 (6)	13 (10)	0.354
CVC, *n* (%)	93 (82)	105 (80)	0.628
Septic shock, *n* (%)	45 (40)	67 (51)	0.096
ICU death, *n* (%)	29 (26)	35 (27)	1.000
OTI duration, median (IQR)	8 (4–13)	7 (3–11)	0.120
MV duration, median (IQR)	8 (3–14)	6 (2–11)	0.044
ICU duration, median (IQR)	11 (6–19)	9 (5–16)	0.078
*Radiographic features*			
Alveolar infiltrates, *n* (%)	72/83*∗* (87)	102/110*∗* (93)	0.223
Unilobar involvement, *n* (%)	36/82*∗* (44)	59/109*∗* (54)	0.189
Multilobar involvement, *n* (%)	35/82*∗* (43)	42/109*∗* (39)	0.655
*Antimicrobial therapy*			
Stream lining (de-escalation), *n* (%)	59 (53)	87 (67)	0.025
De-escalation possibility, *n* (%)	84 (76)	111 (85)	0.102
Outcome			
Death in ICU, *n* (%)	29 (26)	35 (27)	0.996

ARDS = acute respiratory distress syndrome; BMI = body mass index; CAP = community-acquired pneumonia; COPD = chronic obstructive pulmonary disease; CVC = central venous catheter; HAP = hospital-acquired pneumonia; ICU = intensive care unit; IQR = interquartile range; MV = mechanical ventilation; OTI = orotracheal intubation; SAPS-II = Simplified Acute Physiology Score-II; VAP = ventilator-associated pneumonia. *∗*Patients with available data.

**Table 2:** Demographical, microbiological, and clinical characteristics of patients affected by *Haemophilus influenzae* pneumonia according to amoxicillin susceptibility.

Criteria	Amoxicillin susceptible (*n* = 78)	Amoxicillin resistant (*n* = 35)	*p* value
*Demographical characteristics*			
Female gender, *n* (%)	32 (41)	14 (40)	1.000
Age, median (IQR)	62.5 (20–88)	67 (31-92)	0.545
BMI, median (IQR)	25.5 (43-16)	25 (19–42)	0.779
*Medical history*			
Recent bronchitis, *n* (%)	13 (16.6)	8 (22.8)	0.443
Previous pneumonia, *n* (%)	10 (12.8)	5 (14.2)	1.000
*Risk factors*			
Living in nursing home, *n* (%)	3 (3.8)	1 (2.8)	1.000
Smoking, *n* (%)	38 (48.7)	17 (48.5)	1.000
Alcohol abuse, *n* (%)	25 (32)	8 (22.8)	0.376
*Comorbidities*			
Immunosuppression, *n* (%)	10 (12.8)	7 (20)	0.395
Diabetes, *n* (%)	18 (23)	7 (20)	0.809
Cirrhosis, *n* (%)	7 (8.9)	2 (5.7)	0.718
Renal chronic failure, *n* (%)	3 (3.8)	2 (5.7)	0.644
Cardiac chronic failure, *n* (%)	15 (19.2)	6 (17.1)	1.000
COPD, *n* (%)	33 (42.3)	16 (45.7)	0.837
*Type of pneumonia*			
CAP, *n* (%)	65 (83.3)	28 (80)	0.790
HAP, *n* (%)	13 (16.6)	7 (20)	0.790
VAP, *n* (%)	9 (11.5)	6 (17.1)	0.549
Inhalation associated, *n* (%)	33 (42.3)	9 (25.7)	0.098
Coinfection, *n* (%)	23 (29.4)	8 (22.8)	0.504
*Kind of sample*			
Pulmonary sample, *n* (%)	75 (96.2)	31 (88.6)	0.200
Blood culture, *n* (%)	3 (3.8)	4 (11.4)	
Pleural fluid, *n* (%)	0 (0)	0 (0)	
Cerebrospinal fluid, *n* (%)	0 (0)	0 (0)	
*Severity of pneumonia*			
SAPS-II, median (IQR)	48 (14–104)	49 (24–90)	0.806
Local complication, *n* (%)	6 (7.7)	2 (5.7)	1.000
Organ failure, *n* (%)	73 (93.5)	35 (100)	0.321
ARDS, *n* (%)	10 (12.8)	7 (20)	0.395
MV, *n* (%)	74 (94.8)	33 (94.3)	1.000
OTI, *n* (%)	65 (83.3)	31 (88.6)	0.577
VAP during hospitalization, *n* (%)	21 (26.9)	10 (28.5)	1.000
Acute renal failure, *n* (%)	36 (46.1)	19 (54.3)	0.541
Dialysis, *n* (%)	3 (3.8)	4 (11.4)	0.200
CVC, *n* (%)	63 (80.7)	30 (85.7)	0.603
Septic shock, *n* (%)	32 (41)	13 (37.1)	0.680
ICU death, *n* (%)	19 (24.3)	10 (28.5)	0.647
OTI duration, median (IQR)	7 (1–70)	11 (1–41)	0.583
MV duration, median (IQR)	8 (0–70)	10 (0–41)	0.410
ICU duration, median (IQR)	10 (1–74)	12 (2–102)	0.337
*Antimicrobial therapy*			
Stream lining (de-escalation), *n* (%)	40 (51.2)	19 (54.3)	0.840
De-escalation possibility, *n* (%)	58 (74.3)	26 (74.3)	1.000
*Outcome*			
Death in ICU, *n* (%)	19 (24.4)	10 (28.6)	0.635

ARDS = acute respiratory distress syndrome; BMI = body mass index; CAP = community-acquired pneumonia; COPD = chronic obstructive pulmonary disease; CVC = central venous catheter; HAP = hospital-acquired pneumonia; ICU = intensive care unit; IQR = interquartile range; MV = mechanical ventilation; OTI = orotracheal intubation; SAPS-II = Simplified Acute Physiology Score-II; VAP = ventilator-associated pneumonia.

## Data Availability

The data used to support the findings of the study are available from the corresponding author upon request.

## References

[1] Welte T., Torres A., Nathwani D. (2012). Clinical and economic burden of community-acquired pneumonia among adults in Europe. *Thorax*.

[2] Parrott G., Nebeya D., Kinjo T. (2017). Etiological analysis and epidemiological comparison among adult CAP and NHCAP patients in Okinawa, Japan. *Journal of Infection and Chemotherapy*.

[3] Hariri G., Tankovic J., Boëlle P.-Y. (2017). Are third-generation cephalosporins unavoidable for empirical therapy of community-acquired pneumonia in adult patients who require ICU admission? A retrospective study. *Annals of Intensive Care*.

[4] Forstner C., Rohde G., Rupp J. (2016). Community-acquired Haemophilus influenzae pneumonia—new insights from the CAPNETZ study. *Journal of Infection*.

[5] Vila-Corcoles A., Bejarano-Romero F., Salsench E. (2009). Drug-resistance in Streptococcus pneumoniae isolates among Spanish middle aged and older adults with community-acquired pneumonia. *BMC Infectious Diseases*.

[6] Batard E., Javaudin F., Kervagoret E. (2018). Are third-generation cephalosporins associated with a better prognosis than amoxicillin-clavulanate in patients hospitalized in the medical ward for community-onset pneumonia?. *Clinical Microbiology and Infection*.

[7] Gould I. M. (2008). The epidemiology of antibiotic resistance. *International Journal of Antimicrobial Agents*.

[8] Dalhoff A. (2012). Resistance surveillance studies: a multifaceted problem-the fluoroquinolone example. *Infection*.

[9] Kalil A. C., Metersky M. L., Klompas M. (2016). Management of adults with hospital-acquired and ventilator-associated pneumonia: 2016 clinical practice guidelines by the infectious diseases society of America and the American thoracic society. *Clinical Infectious Diseases*.

[10] Metlay J. P., Waterer G. W., Long A. C. (2007). Diagnosis and treatment of adults with community-acquired pneumonia. An official clinical practice guideline of the American thoracic society and infectious diseases society of America. *American Journal of Respiratory and Critical Care Medicine*.

[11] Leclercq R., Cantón R., Brown D. F. J. (2013). EUCAST expert rules in antimicrobial susceptibility testing. *Clinical Microbiology and Infection*.

[12] Deghmane A.-E., Hong E., Chehboub S. (2019). High diversity of invasive Haemophilus influenzae isolates in France and the emergence of resistance to third generation cephalosporins by alteration of ftsI gene. *Journal of Infection*.

[13] European Centre for Disease Prevention and Control, Surveillance atlas of infectious diseases, https://atlas.ecdc.europa.eu/public/index.aspx?Dataset=27&%20Health%20Topic=4

[14] Yamana H., Matsui H., Tagami T., Hirashima J., Fushimi K., Yasunaga H. (2016). De-escalation versus continuation of empirical antimicrobial therapy in community-acquired pneumonia. *Journal of Infection*.

[15] Weiss E., Zahar J.-R., Lesprit P. (2015). Elaboration of a consensual definition of de-escalation allowing a ranking of *β*-lactams. *Clinical Microbiology and Infection*.

[16] Viasus D., Simonetti A. F., Garcia-Vidal C., Niubó J., Dorca J., Carratalà J. (2017). Impact of antibiotic de-escalation on clinical outcomes in community-acquired pneumococcal pneumonia. *Journal of Antimicrobial Chemotherapy*.

[17] Braeken D. C. W., Franssen F. M. E., Von Baum H. (2017). Bacterial aetiology and mortality in COPD patients with CAP: results from the German Competence Network, CAPNETZ. *The International Journal of Tuberculosis and Lung Disease*.

[18] WHO Recommendations for Routine Immunization, https://www.who.int/immunization/policy/immunization_tables/en/, 2019

